# Vitamin D Receptor rs7975232 (*ApaI*) Variant, Inflammatory Markers, and Patient-Reported Outcomes in Orthopedic Surgery

**DOI:** 10.3390/jcm14217675

**Published:** 2025-10-29

**Authors:** Dariusz Larysz, Remigiusz Recław, Aleksandra Suchanecka, Wojciech Dziurawiec, Rafał Tkacz, Aleksandra Strońska-Pluta, Krzysztof Chmielowiec, Anna Grzywacz, Jolanta Chmielowiec

**Affiliations:** 1 Department of Trauma and Orthopedic Surgery, 109th Military Hospital with Polyclinic, Ministry of National Defence, Ksiedza Piotra Skargi 9/11 St., 71-422 Szczecin, Poland; dariuszlarysz@hotmail.com (D.L.); dziurawiec.wojciech@gmail.com (W.D.); rafaltkacz@gmail.com (R.T.); 2 Independent Laboratory of Behavioral Genetics and Epigenetics, Pomeranian Medical University in Szczecin, Powstańców Wielkopolskich 72 St., 70-111 Szczecin, Poland; remigiusz.reclaw@pum.edu.pl (R.R.); aleksandra.suchanecka@pum.edu.pl (A.S.); aleksandra.stronska@pum.edu.pl (A.S.-P.); 3 Department of Medical Sciences and Public Health, Gdansk University of Physical Education and Sport, Kazimierza Górskiego 1 St., 80-336 Gdansk, Poland; 4 Department of Hygiene and Epidemiology, Collegium Medicum, University of Zielona Góra, 28 Zyty St., 65-046 Zielona Góra, Poland; chmiele@vp.pl; 5 Department of Nursing, Collegium Medicum, University of Zielona Góra, 28 Zyty St., 65-046 Zielona Góra, Poland; j.chmielowiec@inz.uz.zgora.pl

**Keywords:** vitamin D receptor (VDR), rs7975232 *ApaI*, orthopedic surgery, quality of life (HRQoL)

## Abstract

**Background:** Genetic variability in the vitamin D receptor (VDR) may influence immune regulation and systemic inflammation, factors potentially relevant for outcomes in orthopedic surgery. This study explored the association of the *VDR* rs7975232 (*ApaI*) single nucleotide variation (SNV) with inflammatory biomarkers and health-related quality of life (HRQoL). **Methods:** The study included 292 orthopedic patients and 90 controls. Genotyping was performed using real-time PCR. Laboratory analyses comprised hematological parameters, C-reactive protein (CRP), and serum 25-hydroxyvitamin D_3_ [25(OH)D_3_]. HRQoL was assessed with the SF-36 questionnaire. Associations between genotype, inflammation, and HRQoL were examined using regression models adjusted for age and body mass index (BMI). **Results:** Genotype (*p* = 0.023) and allele (*p* = 0.007) distributions differed between patients and controls. In multivariable models, the CC genotype was associated with higher neutrophil counts (*p* = 0.029), whereas the AA genotype was associated with elevated CRP levels (*p* = 0.025). Genotypic variation was further associated with SF-36 scores, independently of age and BMI. **Conclusions:** The *VDR* rs7975232 SNV may be associated with baseline systemic inflammation and patient-reported quality of life in orthopedic surgery. Genotyping of this variant may complement conventional biomarkers in future research aimed at improving diagnostic precision. Moreover, it could contribute to innovation in laboratory diagnostics aimed at improving perioperative outcomes.

## 1. Introduction

Vitamin D is essential not only for skeletal integrity through its effects on calcium–phosphate balance but also for broader immunomodulatory functions and the regulation of systemic inflammation [1,2,3,4]. Suboptimal vitamin D status has been consistently linked to impaired musculoskeletal recovery and higher perioperative risk. Beyond serum levels, accumulating evidence suggests that the *VDR* rs7975232 (*ApaI*) single nucleotide variation (SNV) may influence clinically relevant outcomes: fracture risk [5,6], susceptibility to perioperative infections such as osteomyelitis, and health-related quality of life [7,8]. At the same time, meta-analyses indicate that *ApaI* is not considered a consistent risk factor for osteoarthritis [9,10]. Overall, the role of evidence on the *ApaI* SNV across different disorders remains inconsistent, with its impact appearing to depend on clinical context and population characteristics. This variability further underscores the need to specifically evaluate its significance in orthopedic patients, where perioperative outcomes remain a pressing concern. While serum 25-hydroxyvitamin D_3_ [25(OH)D_3_] concentration remains the standard clinical marker, accumulating evidence indicates that genetic variation within the vitamin D receptor (VDR) gene provides additional insights that cannot be captured by serum levels alone [7,11,12,13]. Given the increasing global burden of orthopedic procedures and the substantial healthcare costs related to postoperative complications, understanding molecular determinants of recovery has direct clinical and economic relevance.

The *ApaI* allelic variant (rs7975232) is an intronic variant, and its functional effects are thought to arise primarily through regulation of gene expression at the mRNA level rather than direct alterations of receptor protein structure. Among the most frequently investigated loci, *VDR* rs7975232 (*ApaI*) has been implicated in differential immune responses, variable inflammatory activity, and vulnerability to chronic disease [14,15,16]. Such associations are particularly relevant in the orthopedic setting, where perioperative inflammation not only shapes short-term surgical risk but also influences rehabilitation potential and long-term quality of life. Notably, *ApaI* variation has also been associated with differences in health-related quality of life in non-orthopedic conditions such as primary sclerosing cholangitis [8], suggesting that its influence extends beyond traditional biomedical markers and may capture patient-centered outcomes of high clinical relevance. Building on such evidence, investigating HRQoL in orthopedic cohorts highlights the innovative dimension of the present study. However, despite evidence linking VDR allelic variants to immune modulation [17], the role of rs7975232 in orthopedic patients remains poorly characterized, and its potential clinical utility as a biomarker in perioperative care has not been systematically explored.

This perspective aligns with the ongoing transformation of laboratory medicine, where innovation increasingly involves integrating molecular testing into clinical workflows [11,12]. Advances in single nucleotide variation genotyping have enabled rapid, cost-effective, and widely accessible analyses, creating opportunities to complement traditional biochemical assays such as C-reactive protein (CRP) and complete blood count (CBC) with stable, lifelong genetic information. Such integration reflects a broader paradigm shift from static, population-based diagnostics toward precision medicine, in which molecular data guide personalized care pathways and inform patient stratification [7,17].

The present study investigated the associations of *VDR* rs7975232 (*ApaI*) with systemic inflammatory markers and health-related quality of life (HRQoL) in orthopedic patients. It was hypothesized that variation in rs7975232 would be associated with differences in inflammatory activity and HRQoL outcomes, independent of age and body mass index. The objective was not only to determine whether this variant contributes to patient heterogeneity but also to evaluate its translational value as a potential adjunct to conventional laboratory testing, thereby supporting more accurate perioperative risk assessment and advancing diagnostic innovation in orthopedic care.

### Key Points

- The *VDR* rs7975232 (*ApaI*) variant may be associated with systemic inflammatory markers and patient-reported quality of life.
- Genotyping provides lifelong molecular information that could complement, rather than replace, conventional laboratory markers.
- Future studies may explore whether incorporating *VDR* genotyping could inform individualized perioperative monitoring or rehabilitation strategies.
- This study exemplifies the broader innovation in laboratory medicine, where integration of molecular and biochemical markers drives precision diagnostics and personalized care.

## 2. Materials and Methods

### 2.1. Participants

The study enrolled a total of 382 participants. The patient group consisted of 292 individuals scheduled for orthopedic surgery (112 men, 38%; 180 women, 62%), whereas the control group comprised 90 volunteers without a history of musculoskeletal disorders or prior orthopedic interventions (27 men, 30%; 63 women, 70%, Table 1). The mean age of patients was 66.7 years, compared with 64.5 years in the control group. Surgical patients were recruited from the Orthopedics Department of the 109th Military Hospital with Polyclinic in Szczecin, Poland, and were eligible if they were over 18 years of age, were qualified for knee or hip replacement surgery, qualified for surgical treatment of degenerative changes in the spine, qualified for joint replacement surgery or spinal surgery with stabilization, free from active malignancy, infectious or autoimmune disease, and provided written informed consent. Controls were recruited primarily from the local community, as well as university staff.

### 2.2. Measures

Biochemical analyses were performed for all participants and included the following parameters: white blood cell count (WBC, ×10^9^/L), lymphocyte count (LYM, ×10^9^/L), neutrophil count (NEU, ×10^9^/L), monocyte count (MONO, ×10^9^/L), eosinophil count (EOS, ×10^9^/L), basophil count (BASO, ×10^9^/L), hemoglobin concentration (HGB, g/dL), hematocrit (HCT, %), mean corpuscular volume (MCV, fL), mean corpuscular hemoglobin (MCH, pg), mean corpuscular hemoglobin concentration (MCHC, g/dL), platelet count (PLT, ×10^9^/L), platelet distribution width (PDW, %), mean platelet volume (MPV, fL), plateletcrit (PCT, %), C-reactive protein (CRP, mg/L), serum 25-hydroxyvitamin D_3_ (25(OH)D_3_, ng/mL), glycated hemoglobin (HbA1c, %), and serum creatinine (µmol/L). All blood samples, including CRP and complete blood count, were collected during preoperative assessment, prior to surgical intervention.

Body mass index (BMI, kg/m^2^) was calculated as body weight in kilograms divided by the square of height in meters.

Health-related quality of life was evaluated with the Short Form Health Survey (SF-36), a validated 36-item instrument that measures eight domains: physical functioning, role limitations due to physical health, bodily pain, general health, vitality, social functioning, role limitations due to emotional problems, and mental health.

### 2.3. Genotyping

Genomic DNA was extracted from peripheral venous blood using standardized kit following the manufacturer’s instructions (ROCHE, Basel, Switzerland) and in accordance with the company’s quality control procedures. Reagents, primers, and probes were selected according to the specifications of the ROCHE real-time PCR system for single nucleotide variation analysis.

The vitamin D receptor (*VDR*) rs7975232 *ApaI* (C/A) SNV was genotyped using real-time PCR. After amplification, melting curve analysis was performed for each sample by plotting fluorescence intensity against temperature. Characteristic melting peaks allowed for allele identification: the C allele was detected at approximately 62.8 °C, and the A allele at approximately 69.9 °C. Each run included negative (no-template) controls. Additionally, 10% of randomly selected samples was re-genotyped to confirm reliability.

### 2.4. Statistics

Hardy–Weinberg equilibrium (HWE) for genotype frequencies was assessed using HWE software (https://sites.google.com/view/generiskcalc/home/gene-association-calculator, accessed on 15 October 2025). Normality of variable distribution was verified with the; QQ-plots (Supplementary Materials Figure S1). For data in which QQ-plots showed non-normally distributed variable features, transformations were performed by logarithm normalization (Supplementary Materials Figure S2). Variables with normal distribution are reported as mean ± standard deviation (SD), whereas those with non-normal distribution are presented as median with interquartile range [Q1–Q3].

Comparisons of blood parameters between males and females were carried out using Student’s *t*-test for normally distributed variables and the Mann–Whitney U test for variables not meeting normality assumptions. Categorical variables were compared using the chi-square test. Associations between the rs7975232 *ApaI* sequence variant and biochemical parameters, as well as scores from the SF-36 Quality of Life Questionnaire, were examined with multiple regression models. Dependent variables included blood parameters and SF-36 scores in orthopedic patients, while independent variables were age, body mass index (BMI), and the rs7975232 *ApaI* genotype. For this allelic variant, a dummy coding approach was applied: CA heterozygotes served as the reference group, while homozygotes were used to assess the effect on the dependent variables.

For variables related to biochemical markers included in the Supplement, a significance level of 0.0025 (0.05/20) was set using the Bonferroni correction for multiple comparisons.

A *p*-value < 0.05 was considered statistically significant. Statistical analyses were performed using STATISTICA 13 (Tibco Software Inc., Palo Alto, CA, USA), PQStat version 1.8.6 (Poznań, Poland) and JASP 0.95.2 (University of Amsterdam & others) for Windows 11 (Microsoft Corporation, Redmond, WA, USA).

## 3. Results

The distribution of rs7975232 genotypes in orthopedic patients conformed to Hardy–Weinberg equilibrium (HWE). The control group also showed no deviation from HWE (Table 2).

A statistically significant difference was found in the distribution of rs7975232 *ApaI* genotypes between orthopedic patients and controls (CC 0.25 vs. 0.39; CA 0.52 vs. 0.47; AA 0.23 vs. 0.14; χ^2^ = 7.5778, *p* = 0.02262). A corresponding difference was also noted in allele frequencies between the two groups (C 0.51 vs. 0.62; A 0.49 vs. 0.38; χ^2^ = 7.14573, *p* = 0.00751; Table 3).

No significant differences in rs7975232 *ApaI* genotype distribution were detected among orthopedic patients when stratified by the presence of hypertension (χ^2^ = 1.48468, *p* = 0.47600), or diabetes mellitus (χ^2^ = 4.01995, *p* = 0.13399; Table 4).

The baseline characteristics of orthopedic patients, including age, BMI, biochemical parameters, and SF-36 quality of life scores, are summarized in Table 5. The distribution of variables was verified using QQ-plots (see Supplementary Materials for visualizations). Table 5 provides full names of the parameters together with their abbreviations and units, while only abbreviations are used in Table 6 and Table 7 for clarity.

Table 6 shows sex-related differences in biochemical parameters and SF-36 quality of life scores among orthopedic patients. Compared with women, men demonstrated significantly higher body mass index (29.96 vs. 28.76; t = 1.96907, *p* = 0.04991), monocyte count (0.57 vs. 0.50; t = 3.7940, *p* = 0.00018), hemoglobin concentration (14.49 vs. 13.37; t = 6.9628, *p* < 0.00001), hematocrit (43.02% vs. 40.07%; t = 5.8694, *p* < 0.00001), mean corpuscular volume (91.48 vs. 90.02; t = 2.4084, *p* = 0.01664), mean corpuscular hemoglobin (30.81 vs. 29.79; t = 4.1028, *p* = 0.00005), mean corpuscular hemoglobin concentration (33.68 vs. 33.14; t = 4.7251, *p* < 0.00001), plateletcrit (0.54 vs. 0.36; Z = 3.7583, *p* = 0.00014), and serum creatinine concentration (90.34 μmol/L vs. 73.98 μmol/L; Z = 6.3369, *p* < 0.00001). In contrast, men exhibited significantly lower platelet count (240.47 vs. 267.11; t = −3.1257, *p* = 0.00195) and serum 25-hydroxyvitamin D_3_ concentration (29.02 ng/mL vs. 34.80 ng/mL; Z = −3.2759, *p* = 0.00105).

**Table 6:** Sex-based differences in biochemical parameters and SF-36 quality of life scores among orthopedic patients.

Sex	Men = 112 M (SD)/Me * [Q1:Q3] *	Women = 180 M (SD)/Me * [Q1:Q3] *	Student’s *t*-Test/Mann–Whitney U Test *	*p*-Value
Age	66.09 (9.83)	67.08 (10.38)	−0.8116	0.41767
BMI	29.96 (4.98)	28.76 (5.06)	1.96907	0.04991 #
WBC	7.24 (1.89)	6.82 (2.00)	1.7692	0.0779
LYM	1.73 (0.55)	1.69 (0.58)	0.6586	0.51068
NEU	4.74 (1.63)	4.47 (1.73)	1.323	0.18687
MONO	0.57 (0.18)	0.50 (0.17)	3.794	0.00018 #ǂ
EOS	0.13 [0.06:0.18]	0.12 [0.04:0.16]	1.2863 *	0.19832
BASO	0.04 [0.02:0.05]	0.04 [0.03:0.05]	−0.7247 *	0.46859
HGB	14.49 (1.54)	13.37 (1.20)	6.9628	0.00001 #ǂ
HCT %	43.02 (4.22)	40.07 (4.14)	5.8694	0.00001 #ǂ
MCV	91.48 (5.24)	90.02 (4.92)	2.4084	0.01664 #
MCH	30.81 (2.00)	29.79 (2.11)	4.1028	0.00005 #ǂ
MCHC	33.68 (0.93)	33.14 (0.95)	4.7251	0.00001 #ǂ
PLT	240.47 (60.87)	267.11 (76.33)	−3.1257	0.00195 #ǂ
PDW	14.77 [10.70:13.40]	12.50 [11.10:13.50]	−0.7344 *	0.46266
MPV	10.35 [9.70:11.10]	12.31 [9.80:11.10]	−0.9857 *	0.32425
PCT	0.54 [0.21:0.28]	0.36 [0.23:0.33]	3.7583 *	0.00017 #ǂ
CRP	7.53 [0.85:4.09]	7.81 [1.07:4.83]	−1.0276 *	0.30411
25(OH)D_3_	29.02 [19.10:34.40]	34.80 [22.40:42.90]	−3.2759 *	0.00105 #ǂ
HbA1c	5.68 [5.36:6.03]	5.80 [5.38:5.94]	0.4746 *	0.63505
Creatinine	90.34 [72.00:99.50]	73.98 [61.00:82.00]	6.3369 *	0.00001 #ǂ
SF-36 PCS	46.04 (10.76)	45.43 (10.63)	−0.4467	0.6554
SF-36 MCS	47.98 (15.46)	47.73 (14.57)	−0.1272	0.89892

M (mean); SD (standard deviation); Q1 (lower quartile); Q3 (upper quartile); * (non-normally distributed variable; QQ-plots—Supplementary Materials); Student’s *t*-test (used for normally distributed variables); Mann–Whitney U test (used for non-normally distributed variables, marked *); # (statistically significant difference, *p* < 0.05). ǂ Bonferroni correction was applied, and the *p*-value was lowered to 0.0025.

Table 7 summarizes the results of multiple regression analysis, with rs7975232 *ApaI* genotype, age, and body mass index included as independent variables predicting biochemical parameters and SF-36 quality of life scores in orthopedic patients. Statistically significant model fits were observed for the following outcomes: white blood cell count, lymphocyte count, monocyte count, basophil count, hemoglobin concentration, hematocrit, mean corpuscular volume, platelet count, plateletcrit, C-reactive protein, serum 25-hydroxyvitamin D_3_ (ng/mL), serum creatinine (μmol/L), and SF-36 score.

A higher neutrophil count was associated with the rs7975232 *ApaI* CC genotype (β = 0.53; 95% CI [0.05, 1.00]; *p* = 0.028849). Elevated C-reactive protein was associated with the rs7975232 *ApaI* AA genotype (β = 7.80; 95% CI [0.98, 14.62]; *p* = 0.02504) and log CRP (β = 0.35; 95% CI [0.01, 0.71]; *p* = 0.05716).

Older age was associated with lower lymphocyte count (β = −0.01; 95% CI [−0.02, −0.003]; *p* = 0.00405), lower basophil count (β = −0.0002; 95% CI [−0.0004, −0.00002], *p* = 0.03237) and basophil count (β = −0.0002; 95% CI [−0.0004, −0.00002], *p* = 0.03237), lower platelet count (β = −0.005 [−0.01, 0.0005]; *p* = 0.07499), lower plateletcrit (β = −0.02; 95% CI [−0.04, −0.003]; *p* = 0.02460) and log plateletcrit (β = −0.008; 95% CI [−0.01, −0.003]; *p* = 0.00469). In contrast, older age was associated with higher 25-hydroxyvitamin D_3_ levels (β = 0.32; 95% CI [0.15, 0.50]; *p* = 0.00036) and log 25-hydroxyvitamin D_3_ levels (β = 0.01; 95% CI [0.005, 0.02]; *p* = 0.00079), higher creatinine concentration (β = 0.61; 95% CI [0.31, 0.92] *p* = 0.00010) and log creatinine concentration (β = 0.006; 95% CI [0.003, 0.01]; *p* = 0.00004).

Higher body mass index was associated with increased lymphocyte count (β = 0.01; 95% CI [0.002,0.03]; *p* = 0.01795), monocyte count (β = 0.007; 95% CI [0.003, 0.01]; *p* = 0.00059), eosinophil count (β = 0.003; 95% CI [0.001, 0.005]; *p* = 0.01324) and log eosinophil count (β = 0.03; 95% CI [0.01, 0.05]; *p* = 0.00241), hematocrit (β = 0.15; 95% CI [0.05, 0.25]; *p* = 0.00358), and creatinine concentration (β = 0.70; 95% CI [0.09, 1.30]; *p* = 0.02409) and log creatinine concentration (β = 0.008; 95% CI [0.002, 0.01]; *p* = 0.01179). Conversely, higher BMI was associated with lower 25-hydroxyvitamin D_3_ levels (β = −0.47; 95% CI [−0.82, −0.12]; *p* = 0.00944) and log 25-hydroxyvitamin D_3_ levels (β = −0.01; 95% CI [−0.02, 0.001]; *p* = 0.06461).

**Table 7:** Multiple regression analysis of biochemical parameters and SF-36 quality of life scores with rs7975232 *ApaI* genotype (C/A as reference), age, and body mass index as predictors.

Variable	Reference β [−95%CI, +95%CI] *p* Value	rs7975232 A/A β [−95%CI, +95%CI] *p* Value	rs7975232 C/C β [−95%CI, +95%CI] *p* Value	Age β [−95%CI, +95%CI] *p* Value	BMI β [−95%CI, +95%CI] *p* Value
WBC	5.90 [3.89, 7.91] *p* < 0.00001 #	0.35 [−0.21, 0.91] *p* = 0.22858	0.43 [−0.12, 0.98] *p* = 0.12468	−0.004 [−0.03, 0.02] *p* = 0.74017	0.04 [−0.01, 0.08] *p* = 0.09360
LYM	1.91 [1.33, 2.49] *p* < 0.00001 #	−0.07 [−0.23, 0.09] *p* = 0.39711	−0.06 [−0.22, 0.10] *p* = 0.45858	−0.01 [−0.02, −0.003] *p* = 0.00405 #	0.01 [0.002, 0.03] *p* = 0.01795 #
NEU	3.50 [1.78, 5.21] *p* = 0.00008 #	0.42 [−0.07, 0.90] *p* = 0.09243	0.53 [0.05, 1.00] *p* = 0.028849 #	0.007 [−0.01, 0.03] *p* = 0.50463	0.01 [−0.03, 0.05] *p* = 0.49141
MONO	0.31 [0.13, 0.48] *p* = 0.00065 #	−0.003 [−0.05, 0.05] *p* = 0.91245	−0.03 [−0.07, 0.02] *p* = 0.28169	0.0003 [−0.002, 0.002] *p* = 0.73708	0.007 [0.003, 0.01] *p* = 0.00059 #
EOS	0.03 [−0.07, 0.13] *p* = 0.58019	0.01 [−0.02, 0.04] *p* = 0.49614	−0.005 [−0.03, 0.02] *p* = 0.72198	0.0001 [−0.001, 0.001] *p* = 0.89897	0.003 [0.001, 0.005] *p* = 0.01324 #
log EOS *	−3.36 [4.23, −2.49] *p* = 0.00001 #	0.10 [−0.15, 0.35] *p* = 0.42711	−0.08 [−0.31, 0.16] *p* = 0.518327	0.001 [−0.01, 0.01] *p* = 0.82238	0.03 [0.01, 0.05] *p* = 0.00241 #
BASO	0.04 [0.02, 0.06] *p* = 0.00001 #	0.002 [−0.003, 0.007] *p* = 0.47394	−0.002 [−0.007, 0.003] *p* = 0.46312	−0.0002 [−0.0004, −0.00002] *p* = 0.03237 #	0.0003 [−0.0001, 0.0007] *p* = 0.13294
log BASO *	−3.40 [−3.91, −2.89] *p* = 0.00001 #	0.03 [−0.11, 0.18] *p* = 0.66737	−0.06 [−0.20, 0.08] *p* = 0.37578	−0.005 [−0.01, 0.0005] *p* = 0.07499	0.01 [−0.001, 0.02] *p* = 0.06505
HGB	13.97 [12.50, 15.45] *p* < 0.00001 #	0.02 [−0.39, 0.44] *p* = 0.92756	−0.04 [−0.45, 0.36] *p* = 0.8449	−0.03 [−0.03, 0.001] *p* = 0.06171	0.03 [−0.003, 0.06] *p* = 0.07303
HCT %	38.86 [34.37, 43.36] *p* = <0.00001 #	−0.04 [−1.31, 1.23] *p* = 0.94957	0.21 [−1.03, 1.44] *p* = 0.74217	−0.03 [−0.08, 0.02] *p* = 0.22091	0.15 [0.05, 0.25] *p* = 0.00358 #
MCV	88.19 [82.91, 93.46] *p* < 0.00001 #	0.27 [−1.21, 1.76] *p* = 0.71773	0.62 [−0.83, 2.07] *p* = 0.40239	0.05 [−0.01, 0.11] *p* = 0.09445	−0.04 [−0.16, 0.08] *p* = 0.50773
MCH	30.12 [27.90, 32.34] *p* < 0.00001 #	−0.03 [−0.65, 0.60] *p* = 0.93714	0.09 [−0.52, 0.70] *p* = 0.77402	0.01 [−0.01, 0.04] *p* = 0.38494	−0.02 [−0.07, 0.03] *p* = 0.34984
MCHC	34.19 [33.19, 35.19] *p* < 0.00001 #	−0.18 [−0.46, 0.10] *p*= 0.20618	−0.20 [−0.47, 0.08] *p* = 0.16281	−0.01 [−0.02, 0.004] *p* = 0.20587	−0.01 [−0.03, 0.01] *p* = 0.41115
PLT	332.95 [258.96, 406.95] *p* < 0.00001 #	3.54 [−17.33, 24.42] *p* = 0.73856	7.21 [−13.12, 27.54] *p* = 0.485495	−1.11 [−1.94, −0.28] *p* = 0.00868 #	−0.15 [−1.80, 1.51] *p* = 0.85741
PDW	12.94 [−5.61, 31.49] *p* = 0.17071	4.06 [−1.16, 9.29] *p* = 0.12683	−0.10 [−5.19, 4.99] *p* = 0.96901	−0.09 [−0.30, 0.12] *p* = 0.38427	0.19 [−0.22, 0.61] *p* = 0.35570
log PDW *	2.41 [2.15, 2.68] *p* < 0.00001 #	0.03 [−0.04, 0.11] *p* = 0.35934	0.01 [−0.06, 0.09] *p* = 0.70598	−0.001 [−0.004, 0.002] *p* = 0.56422	0.005 [−0.001, 0.01] *p* = 0.10452
MPV	16.67 [−3.18, 36.51] *p*= 0.09942	−2.26 [−7.84, 3.33] *p* = 0.42751	−2.10 [−7.54, 3.34] *p* = 0.44835	−0.05 [−0.28, 0.17] *p* = 0.64328	−0.02 [−0.46, 0.43] *p* = 0.93623
log MPV *	2.32 [2.09, 2.55] *p* < 0.00001 #	−0.03 [−0.09, 0.04] *p* = 0.40924	−0.01 [−0.07, 0.05] *p* = 0.69332	−0.00004 [−0.003, 0.003] *p* = 0.97259	0.002 [−0.004, 0.007] *p* = 0.54348
PCT	2.30 [0.62, 3.99] *p* = 0.00753 #	−0.10 [−0.57, 0.37] *p* = 0.66561	0.21 [−0.67, 0.25] *p* = 0.37789	−0.02 [−0.04, −0.003] *p*= 0.02460 #	−0.01 [−0.05, 0.03] *p* = 0.53566
log PCT *	−0.75 [−1.25, −0.25] *p* = 0.00343 #	0.0006 [−0.14, 0.14] *p* = 0.99351	0.003 [−0.14, 0.13] *p* = 0.96569	−0.008 [−0.01, −0.003] *p*= 0.00469 #	0.00003 [−0.01, 0.01] *p* = 0.99618
CRP	8.86 [−15.29, 33.02] *p* = 0.47084	7.80 [0.98, 14.62] *p*= 0.02504 #	−2.03 [−8.67, 4.61] *p* = 0.54765	0.09 [−0.18, 0.37] *p* = 0.49114	−0.30 [−0.84, 0.24] *p* = 0.27446
log CRP *	0.49 [−0.79, 1.78] *p* = 0.45319	0.35 [0.01, 0.71] *P* = 0.05716	−0.07 [−0.42, 0.28] *p* = 0.69010	−0.002 [−0.02, 0.01] *p* = 0.8168	0.02 [−0.01, 0.04] *p* = 0.30056
25(OH)D_3_	23.88 [8.19, 39.57] *p* = 0.00298 #	−1.58 [−6.02, 2.86] *p*= 0.48440	2.25 [−2.05, 6.56] *p* = 0.30261	0.32 [0.15, 0.50] *p* = 0.00036 #	−0.47 [−0.82, −0.12] *p* = 0.00944 #
log 25(OH)D3 *	2.95 [2.38, 3.51] *p* < 0.00001 #	−0.05 [−0.21, 0.11] *p*= 0.52645	0.10 [−0.05, 0.26] *p* = 0.19874	0.01 [0.005, 0.02] *p* = 0.00079 #	−0.01 [−0.02, 0.001] *p* = 0.06461
HbA1c	4.09 [2.26, 5.93] *p* = 0.00002 #	0.08 [−0.44, 0.60] *p* = 0.75801	0.16 [−0.35, 0.66] *p* = 0.5405	0.01 [−0.01, 0.03] *p*= 0.33254	0.03 [−0.01, 0.07] *p* = 0.13255
log HbA1c *	1.55 [0.79, 2.31] *p* = 0.00007 #	−0.05 [−0.27, 0.16] *p* = 0.63397	−0.11 [−0.32, 0.10] *p* = 0.29740	−0.00001 [−0.01, 0.01] *p*= 0.99850	0.005 [−0.01, 0.02] *p* = 0.58861
Creatinine	17.37 [−9.88, 44.62] *p* = 0.21062	4.88 [−2.80, 12.57] *p* = 0.21195	1.18 [−6.21, 8.59] *p* = 0.75317	0.61 [0.31, 0.92] *p* = 0.00010 #	0.70 [0.09, 1.30] *p* = 0.02409 #
log Creatinine *	3.66 [3.39, 3.94] *p* < 0.00001 #	0.07 [−0.01, 0.14] *p* = 0.08968	0.01 [−0.06, 0.09] *p* = 0.70462	0.006 [0.003, 0.01] *p* = 0.00004 #	0.008 [0.002, 0.01] *p* = 0.01179 #
SF−36 PCS	50.96 [39.13, 62.80] *p* < 0.00001 #	1.27 [−2.13, 4.67] *p* = 0.46216	0.13 [−3.08, 3.34] *p* = 0.93617	−0.02 [−0.15, 0.11] *p* = 0.73426	−0.14 [−0.42, 0.13] *p* = 0.30744
SF−36 MCS	45.67 [29.19, 62.15] *p* < 0.00001 #	1.80 [−2.94, 6.53] *p* = 0.45565	4.01 [−0.46, 8.48] *p* = 0.07875	−0.02 [−0.20, 0.16] *p* = 0.84693	0.07 [−0.32, 0.45] *p* = 0.73201

Reference (reference category in the regression model); β (regression coefficient); CI (confidence interval, −95% CI; +95% CI); *p* (statistical significance level); # (statistically significant difference, *p* < 0.05); * transformations by logarithm normalization.

**Figure 1:**
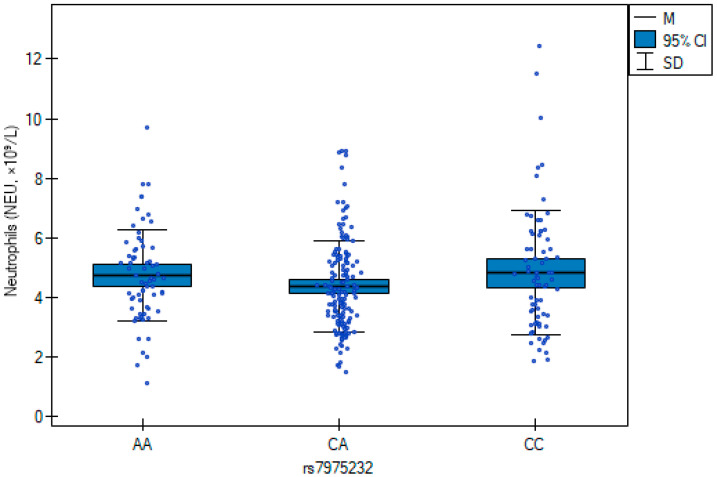
Neutrophil counts across rs7975232 (*ApaI*) genotypes in orthopedic patients. Box plots represent median, interquartile range, and 95% confidence intervals. The CC genotype was associated with higher neutrophil counts compared with CA and AA carriers (β = 0.53; *p* = 0.029).

**Figure 2:**
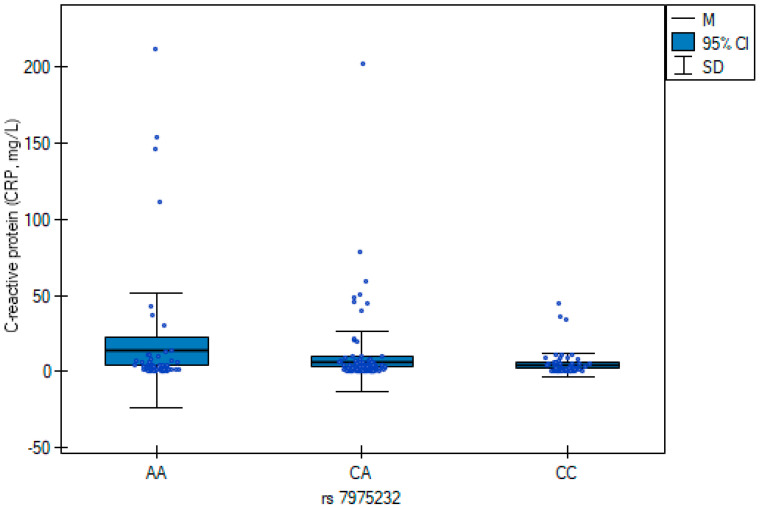
C-reactive protein (CRP) concentrations across rs7975232 (*ApaI*) genotypes in orthopedic patients. Box plots show median values, interquartile ranges, and 95% confidence intervals for each genotype group (AA, CA, CC). Patients with the AA genotype exhibited significantly higher CRP levels compared with CA and CC carriers (β = 7.80; *p* = 0.025).

The results of the multiple regression analysis (Table 7) are illustrated in Figure 1 and Figure 2, showing genotype-related differences in neutrophil counts and C-reactive protein concentrations. CC carriers exhibited higher neutrophil counts, whereas the AA genotype was associated with elevated CRP.

## 4. Discussion

In this study, we explored the association between the vitamin D receptor (*VDR*) rs7975232 (*ApaI*) SNV, systemic inflammatory markers, and patient-reported quality of life in individuals undergoing orthopedic surgery. Specifically, the AA genotype was linked to higher C-reactive protein concentrations, whereas the CC genotype was associated with elevated neutrophil counts. However, we need to emphasize that CRP reflects baseline subclinical inflammation, not acute postoperative changes, and its elevation in AA carriers may indicate a pro-inflammatory genotype-associated phenotype. The graphical representation in Figure 1 and Figure 2 confirmed these regression findings, highlighting distinct genotype-associated inflammatory profiles. Beyond laboratory findings, genotype variation also contributed to differences in health-related quality of life as measured by the SF-36 questionnaire. These results suggest a potential biological link between VDR-related pathways, inflammation, and postoperative well-being.

However, given the cross-sectional and exploratory design, these findings should be interpreted cautiously and not as evidence of causal or predictive relationships.

Previous studies investigating the role of vitamin D in musculoskeletal health have largely focused on circulating 25(OH)D_3_ concentrations as predictors of surgical outcomes, fracture healing, or systemic inflammation. While serum vitamin D deficiency has consistently been linked to poorer recovery trajectories, fewer investigations have examined genetic variation within the *VDR* gene as an additional layer of biological modulation. Among the commonly studied loci, rs2228570 (*FokI*) and rs731236 (*TaqI*) have been associated with bone mineral density, immune regulation, and inflammatory responses, though findings have often been heterogeneous across populations. In contrast, evidence on rs7975232 (*ApaI*) remains limited. Notably, the literature on *ApaI* is inconsistent across clinical contexts: meta-analyses indicate no significant association with osteoarthritis [9,10], in contrast to its reported links with osteoporosis-related traits and fracture risk [5,6]. In the setting of COVID-19, studies have also produced divergent results, with one report suggesting that the AA genotype was more prevalent among deceased patients [18], whereas others found no significant association with disease severity or long-COVID manifestations [19]. These discrepancies underscore the context-dependent nature of *ApaI* effects and emphasize the need for condition-specific evaluation, as pursued in the present orthopedic cohort.

Mechanistically, VDR acts as a nuclear transcription factor [14] that binds to vitamin D response elements in the promoter regions of numerous genes involved in immune regulation [20]. This mechanism is particularly relevant for the *ApaI* allelic variant, which is an intronic variant; its phenotypic effects are presumed to arise from altered mRNA stability and gene expression rather than direct changes in protein structure. This distinguishes *ApaI* from variants such as *FokI*, which generate a shorter but more transcriptionally active VDR isoform [4,11]. Variants such as rs7975232 may therefore influence the balance of pro- and anti-inflammatory gene expression, leading to measurable differences in systemic markers such as CRP and neutrophil count, as well as downstream effects on recovery.

The association with HRQoL may be partly explained through psychoneuroimmunological pathways. Chronic low-grade inflammation has been shown to influence fatigue, mood regulation, and social functioning, which are domains captured by the SF-36 [7]. Variants such as *ApaI*, by modulating inflammatory tone and cytokine signaling, could therefore indirectly affect subjective well-being and perceived recovery. These findings are consistent with prior evidence that vitamin D signaling through VDR modulates both innate and adaptive immunity [21]. Experimental studies have demonstrated that VDR activity can suppress pro-inflammatory cytokines such as TNF-α and IL-6, while enhancing anti-inflammatory mediators, thereby shaping the overall inflammatory milieu [22]. Moreover, VDR influences the differentiation and function of key immune cell populations, including macrophages and T lymphocytes, which are central to postoperative immune responses [23]. In this context, *ApaI*-related differences in CRP and neutrophil counts may reflect upstream modulation of cytokine networks and immune cell activity, linking genetic variation to systemic inflammatory tone and recovery potential.

Genetic variability within *VDR* can provide diagnostic information that extends beyond circulating vitamin D levels by capturing inter-individual differences in inflammatory set points and recovery potential. In the present material, rs7975232 differentiated both inflammatory profiles and patient-reported outcomes, indicating that receptor-level markers can complement the signal obtained from first-line laboratory testing. This receptor-centric perspective is consistent with prior observations in related *VDR* variants, where differences in the gene—not serum 25(OH)D_3_ per se—better accounted for phenotypic heterogeneity [8].

Although preliminary, our findings suggest that *VDR* rs7975232 genotyping could be further investigated as a valuable biomarker in the orthopedic setting. Unlike conventional markers such as CRP or leukocyte count, which fluctuate with acute illness, surgical stress, or transient infections, genetic variation is stable throughout life and requires testing only once. This stability indicates possible translational potential, but further studies are needed to verify whether *VDR* sequence variants provide clinically meaningful information beyond traditional inflammatory markers. For example, *ApaI* and related variants have been shown to influence response to antiresorptive therapy in osteoporosis [5] and to predict treatment outcomes in patients with immune thrombocytopenia treated with corticosteroids [3]. Such evidence illustrates how a lifelong genetic marker can potentially inform research into precision medicine strategies. In orthopedic care, preoperative identification of rs7975232 genotypes could inform clinicians about a patient’s predisposition to heightened inflammatory activity or reduced quality of life after surgery. Importantly, this approach is not intended to replace conventional diagnostics but to complement them. When combined with dynamic biochemical markers, genetic data could provide a more comprehensive risk profile that integrates both current physiological status and inherent biological susceptibility. This dual-layered model aligns with the goals of precision medicine, where static genetic information enriches the interpretation of laboratory results and supports individualized perioperative care.

From a clinical research perspective, future studies may explore whether incorporating *VDR* rs7975232 genotyping into perioperative assessment could help personalize care pathways. If replicated in larger and prospective cohorts, such information might inform individualized approaches to monitoring inflammation and recovery. At this stage, however, these possibilities remain hypothesis-generating rather than clinically actionable. Notably, rs7975232 has also been implicated in disease susceptibility and immune modulation in other autoimmune and inflammatory diseases such as autoimmune thyroiditis [24] and spondyloarthritis [25], further supporting its clinical relevance. Importantly, combining genetic data with patient-reported outcomes (e.g., SF-36 scores) emphasizes the dual relevance of biological and experiential dimensions of recovery. This integration underscores how molecular diagnostics can foster truly patient-centered approaches in orthopedic surgery.

In some contexts, biomarkers and HRQoL measures may diverge in directionality, reflecting overlapping biological and psychosocial components of recovery [26]. This pattern, also noted in prior analyses of related VDR variants, argues for domain-level interpretation of the SF-36 (physical versus mental components) rather than reliance on a single composite score [27], and for prudent interpretation of “hard” biomarkers without clinical context. Such dispersion of signal further justifies combining dynamic markers (CRP, leukogram) with a static genetic fingerprint (*VDR* genotype). By linking molecular markers with both biological and patient-reported outcomes, this study illustrates how laboratory diagnostics can be leveraged to better understand recovery dynamics and the lived experience of patients after surgery.

The potential integration of *VDR* rs7975232 testing into routine laboratory workflows exemplifies the broader trend of innovation in clinical diagnostics [24]. Advances in real-time PCR and genotyping platforms have made single nucleotide variants detection rapid, reliable, and increasingly affordable, allowing results to be generated within hours [28]. This technical progress raises the possibility of incorporating genetic testing into near-patient or point-of-care laboratory settings, where it could complement conventional assays such as complete blood count or CRP. Such integration exemplifies the ongoing innovation in laboratory medicine, where molecular assays extend the diagnostic landscape beyond conventional biochemistry, directly supporting ongoing efforts to advance diagnostic precision in perioperative medicine. In the context of orthopedic surgery, such innovations could transform preoperative screening by enabling clinicians to identify patients genetically predisposed to heightened inflammation or poorer recovery outcomes, and to intervene accordingly. By shifting from a purely reactive model of care toward a predictive and personalized strategy, molecular diagnostics like *VDR* rs7975232 genotyping illustrate how laboratory medicine can directly improve patient outcomes [29]. Practically, a once-only, low-cost genotyping result could be embedded alongside routine panels, providing a stable, lifelong “fingerprint” that could complement dynamic physiological assessments [30]. Although CRP is a non-specific marker, its preoperative elevation is a recognized indicator of baseline inflammatory burden and has been prospectively associated with poorer recovery after joint arthroplasty. The concordant association of rs7975232 with both CRP and neutrophil count strengthens the biological relevance of this finding.

Several limitations of the present study should be acknowledged. First, the analysis focused solely on the rs7975232 allelic variant; inclusion of additional *VDR* variants such as rs2228570 (*FokI*) or rs731236 (*TaqI*) might have provided a more comprehensive view of the receptor’s genetic influence on inflammation and recovery. Second, vitamin D supplementation status and dietary intake were not systematically assessed, which may have modified both biochemical and clinical outcomes. Third, the findings are based on a single-center cohort of Polish patients, limiting the generalizability to other populations and ethnic groups. Finally, although associations with quality of life were observed, causal relationships between genotype, inflammation, and patient-reported outcomes cannot be definitively established within this cross-sectional design.

Additional considerations for future studies include the following: systematic recording of seasonality and supplementation; expansion of the panel to 1,25(OH)_2_D, vitamin D binding protein (DBP), free/bioavailable 25(OH)D_3_, and key cytokines (e.g., IL-6, TNF-α); domain-specific analyses of the SF-36; sensitivity checks using rank-based/robust models for non-normal variables; rigorous control of multiple comparisons; and multi-center, multi-ethnic replication within homogeneous surgical indications. Future studies should also more systematically account for vitamin D supplementation status and serum 25(OH)D_3_ levels, as both factors can modulate the impact of *VDR* SNVs on inflammatory activity and clinical outcomes [4]. Expanding the analysis to include additional VDR variants such as *FokI* or *TaqI* would provide a more comprehensive understanding of receptor-mediated effects and capture polygenic influences [11].

Taken together, our findings provide preliminary evidence for a possible link between the *VDR* rs7975232 allelic variant, inflammatory markers, and patient-reported outcomes. These associations should be viewed as exploratory and hypothesis-generating, requiring replication in larger, multicenter, and longitudinal studies before any clinical application can be considered. Nevertheless, this study contributes novel data on the intersection of molecular genetics and patient-centered outcomes in orthopedic care, encouraging further research into genotype–phenotype interactions within perioperative medicine. Importantly, this study is among the first to link *ApaI* variation with health-related quality of life in orthopedic patients, underscoring the value of integrating patient-reported outcomes alongside molecular and biochemical markers.

## 5. Conclusions

This study provides preliminary evidence that the *VDR* rs7975232 (*ApaI*) single nucleotide variation may be associated with systemic inflammation and patient-reported quality of life in orthopedic patients. By extending the diagnostic perspective on vitamin D biology beyond serum 25(OH)D_3_ levels, our findings suggest the potential to highlight the value of integrating genetic data with conventional laboratory markers. Such integration provides a more comprehensive view of both biological and experiential dimensions of recovery. From a translational perspective, future studies may explore whether, from a clinical standpoint, incorporating *VDR* genotyping into perioperative assessment could refine risk stratification, enable targeted monitoring of inflammatory responses, and support individualised rehabilitation strategies.

The feasibility of rapid and cost-effective PCR-based genotyping platforms translates near-patient laboratory workflows increasingly realistic. Embedding once-only, lifelong genetic information alongside dynamic biochemical markers exemplifies how molecular diagnostics can complement and strengthen routine practice. While replication in larger, multi-centre, and ethnically diverse cohorts remains essential, the present study underscores how innovations in laboratory medicine—specifically, the adoption of genetic biomarkers—can contribute to improving diagnostic precision and potentially inform patient outcomes in orthopedic care.

## Figures and Tables

**Table 1:** Demographic and pharmacotherapy information in patients and controls.

Variable	Patients Undergoing Orthopedic Surgery; n = 292	Control; n = 90	Student’s *t*-Test/χ^2^ *	*p*-Value
Sex Men/Women	112/180 (38.4%/61.6%)	27/63 (30%/70%)	2.0753	0.14970
Age (±SD)	66.70 (±10.17)	64.53 (±13.66)	1.6225	0.1055
BMI (±SD)	29.22 (±5.05)	28.21 (±4.60)	1.6839	0.09303
Smoking status (yes%/no%)	31/261 (10.6%/89.4%)	15/75 (16.7%/83.3%)	2.3776 *	0.12309
Hypertension (yes%/no%)	193/99 (66.1%/33.9%)	65/25 (72.2%/27.8%)	1.7774 *	0.27782
Diabetes mellitus (yes%/no%)	57/235 (19.5%/80.5%)	21/69 (23.3%/76.7%)	0.61547 *	0.43274
Hypertension medications (yes%/no%)	180/112 (62%/38%)	51/39 (57%/43%)	0.71296 *	0.39846
Antidiabetic medications (yes%/no%)	60/232 (21%/79%)	19/71 (21%/79%)	0.01330 *	0.98818
Thyroid diseases medications (yes%/no%)	50/242 (17%/83%)	8/82 (9%/91%)	3.62226 *	0.05701
Cholesterol-lowering medications (yes%/no%)	70/222 (24%/76%)	16/74 (18%/82%)	1.51341 *	0.21862
Anti-reflux medications (yes%/no%)	40/252 (14%/86%)	17/73 (19%/81%)	1.45985 *	0.22695
Asthma/COPD medications (yes%/no%)	15/277 (5%/95%)	2/88 (2%/98%)	1.37453 *	0.24103
Anti-depression/psychiatric disorders medications (yes%/no%)	20/272 (7%/93%)	0/90 (0%/100%)	6.50496 *	0.01076
Anticoagulant therapy (yes%/no%)	40/252 (14%/86%)	10/80 (11%/89%)	0.40490 *	0.52457
Anti-Rheumatologic/inflammatory diseases medications (yes%/no%)	25/267 (9%/91%)	0/90 (0%/100%)	8.24508 *	0.00409

*p*-value (statistical significance, χ^2^ test). * the chi-square test was used.

**Table 2:** Hardy–Weinberg equilibrium analysis of rs7975232 *ApaI* genotype distribution in orthopedic patients and controls.

rs7975232 *ApaI*		Observed (Expected)	Allele freq	χ^2^ *p* Value
Patients undergoing orthopedic surgery; n = 292	CC	73 (75.5)	p (C) = 0.51 q (A) = 0.49	0.3486; *p* = 0.5549
	CA	151 (146.0)		
	AA	68 (70.5)		
Control; n = 90	CC	35 (34.8)	p (C) = 0.62 q (A) = 0.38	0.0049; *p* = 0.9444
	CA	42 (42.3)		
	AA	13 (12.8)		

*p* (statistical significance, χ^2^ test).

**Table 3:** Distribution of rs7975232 *ApaI* variants in orthopedic patients compared with controls.

	Patients Undergoing Orthopedic Surgery; n = 292	Control; n = 90	χ^2^; *p* Value
CC n (%)	73 (25.00%)	35 (38.89%)	7.5778; *p* = 0.02262
CA n (%)	151 (51.71%)	42 (46.67%)	
AA n (%)	68 (23.29%)	13 (14.44%)	
C n (%)	297 (50.86%)	112 (62.22%)	7.14573; *p* = 0.00751
A n (%)	287 (49.14%)	68 (37.78%)	

*p* (statistical significance, χ^2^ test).

**Table 4:** rs7975232 *ApaI* genotype frequencies in orthopedic patients stratified by the presence of hypertension, and diabetes mellitus.

	Men = 112	Women = 180	χ^2^; *p* Value
Hypertension	Yes = 193	No = 99	1.48468; *p* = 0.47600
CC n (%)	46 (23.83%)	27 (27.27%)	
CA n (%)	98 (50.78%)	53 (53.54%)	
AA n (%)	49 (25.39%)	19 (19.19%)	
Diabetes mellitus	Yes = 57	No = 235	4.01995; *p* = 0.13399
CC n (%)	12 (21.05%)	61 (25.96%)	
CA n (%)	26 (45.61%)	125 (53.19%)	
AA n (%)	19 (33.33%%)	49 (20.85%)	

*p* (statistical significance, χ^2^ test).

**Table 5:** Summary of biochemical parameters and SF-36 quality of life scores in orthopedic patients.

	M (SD)/Me * [Q_1_:Q_3_] *	Range (Min–Max)
Age	66.70 (10.08)	27.00–87.00
Body mass index (BMI)	29.22 (5.05)	17.93–48.42
White blood cells (WBC, ×10^9^/L)	6.98 (1.97)	2.40–14.76
Lymphocytes (LYM, ×10^9^/L)	1.70 (0.57)	0.60–4.42
Neutrophils (NEU, ×10^9^/L)	4.57 (1.69)	1.13–12.44
Monocytes (MONO, ×10^9^/L)	0.53 (0.17)	0.05–1.23
Eosinophils (EOS, ×10^9^/L)	0.09 * [0.05:0.17] *	0.00–0.73
Basophils (BASO, ×10^9^/L)	0.03 * [0.02:0.05] *	0.00–0.10
Hemoglobin (HGB, g/dL)	13.80 (1.44)	9.10–17.90
Hematocrit (HCT, %)	41.20 (4.40)	14.70–52.80
Mean corpuscular volume (MCV, fL)	90.58 (5.08)	76.50–120.30
Mean corpuscular hemoglobin (MCH, pg)	30.18 (2.13)	20.80–41.00
Mean corpuscular hemoglobin concentration (MCHC, g/dL)	33.35 (0.97)	28.40–36.60
Platelet count (PLT, ×10^9^/L)	256.89 (71.87)	12.00–717.00
Platelet distribution width (PDW, %)	11.90 * [10.90:13.50] *	8.70–36.9
Mean platelet volume (MPV, fL)	10.40 * [9.80:11.10] *	8.60–14.7
Plateletcrit (PCT, %)	0.26 * [0.22:0.31] *	0.09–00.68
C-reactive protein (CRP, mg/L)	2.01 * [0.99:4.49] *	0.05–211.55
25-hydroxyvitamin D_3_ (ng/mL)	28.85 * [20.40:40.70] *	0.35–101.00
Glycated hemoglobin (HbA1c, %)	5.63 * [5.38:6.00] *	4.72–9.85
Creatinine (µmol/L)	73.50 * [65.00:86.50] *	46.00–282.00
SF-36 PCS	45.67 (10.67)	16.37–76.08
SF-36 MCS	47.83 (14.89)	6.69–94.6

M (mean); SD (standard deviation); Q1 (lower quartile); Q3 (upper quartile); * (non-normally distributed variable, QQ-plots).

## Data Availability

The data presented in this study are available on request from the corresponding author. The data are not publicly available due to privacy concerns.

## Supplementary Materials

The following supporting information can be downloaded at: https://www.mdpi.com/article/10.3390/jcm14217675/s1, Figure S1. QQ-plots of normally distributed variables. QQ-plots normally distributed variable: White blood cells (WBC, ×10^9^/L), Lymphocytes (LYM, ×10^9^/L), Neutrophils (NEU, ×10^9^/L), Monocytes (MONO, ×10^9^/L), Eosinophils (EOS, ×10^9^/L), Basophils (BASO, ×10^9^/L), Hemoglobin (HGB, g/dL), Hematocrit (HCT, %), Mean corpuscular volume (MCV, fL), Mean corpuscular hemoglobin (MCH, pg), Mean corpuscular hemoglobin concentration (MCHC, g/dL), Platelet count (PLT, ×10^9^/L), Platelet distribution width (PDW, %), Mean platelet volume (MPV, fL), Plateletcrit (PCT, %), C-reactive protein (CRP, mg/L), 25-hydroxyvitamin D3 (ng/mL), Glycated hemoglobin (HbA1c, %), Creatinine (µmol/L), SF-36 quality of life score; Figure S2. QQ-plots of variables after logarithmic transformation. QQ-plots normally distributed variable, transformations by logarithm normalization: log Eosinophils (EOS, ×10^9^/L), log Basophils (BASO, ×10^9^/L), log Platelet distribution width (PDW, %), log Mean platelet volume (MPV, fL), log Plateletcrit (PCT, %), log C-reactive protein (CRP, mg/L), log 25-hydroxyvitamin D3 (ng/mL), log Glycated hemoglobin (HbA1c, %), log Creatinine (µmol/L).
