# Supplementary material for: Vitamin D Receptor rs7975232 (ApaI) Variant, Inflammatory Markers, and Patient-Reported Outcomes in Orthopedic Surgery
Source: J Clin Med. 2025 Oct 29;14(21):7675. doi: 10.3390/jcm14217675 (PMC12609321; doi:10.3390/jcm14217675)
Supplement: Supplementary file 1 [file jcm-14-07675-s001.zip › jcm-3897477-supplementary.pdf]

Supplement Materials

QQ-plots were used to visually assess the normality of variable distributions before and after log transformation

Figure S1. QQ-plots of normally distributed variables.

QQ-plots normally distributed variable: White blood cells (WBC, $\times 10^9$/L), Lymphocytes (LYM, $\times 10^9$/L), Neutrophils (NEU, $\times 10^9$/L), Monocytes (MONO, $\times 10^9$/L), Eosinophils (EOS, $\times 10^9$/L), Basophils (BASO, $\times 10^9$/L), Hemoglobin (HGB, g/dL), Hematocrit (HCT, %), Mean corpuscular volume (MCV, fL), Mean corpuscular hemoglobin (MCH, pg), Mean corpuscular hemoglobin concentration (MCHC, g/dL), Platelet count (PLT, $\times 10^9$/L), Platelet distribution width (PDW, %), Mean platelet volume (MPV, fL), Plateletcrit (PCT, %), C-reactive protein (CRP, mg/L), 25-hydroxyvitamin D3 (ng/mL), Glycated hemoglobin (HbA1c, %), Creatinine (µmol/L), SF-36 quality of life score.

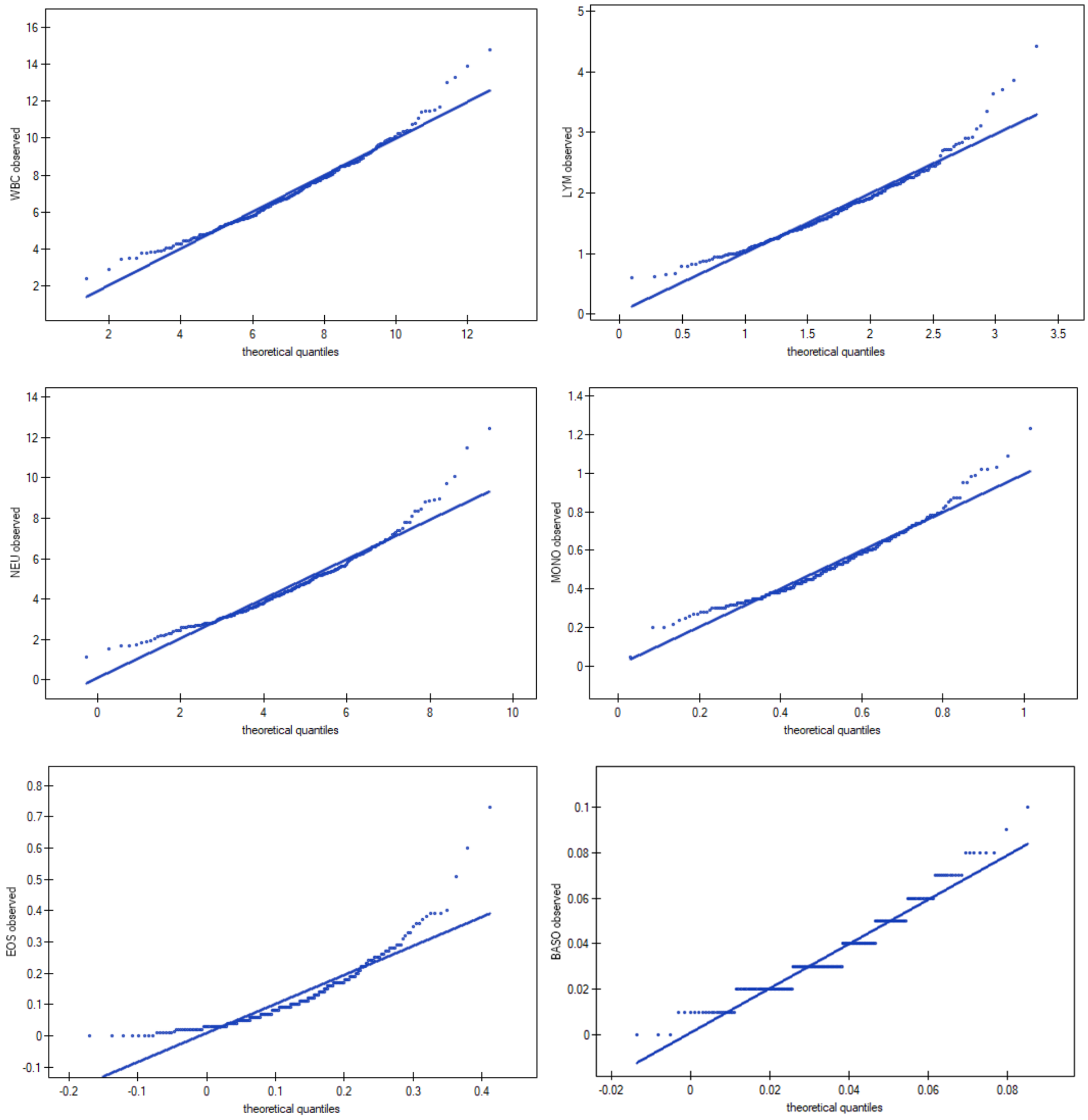

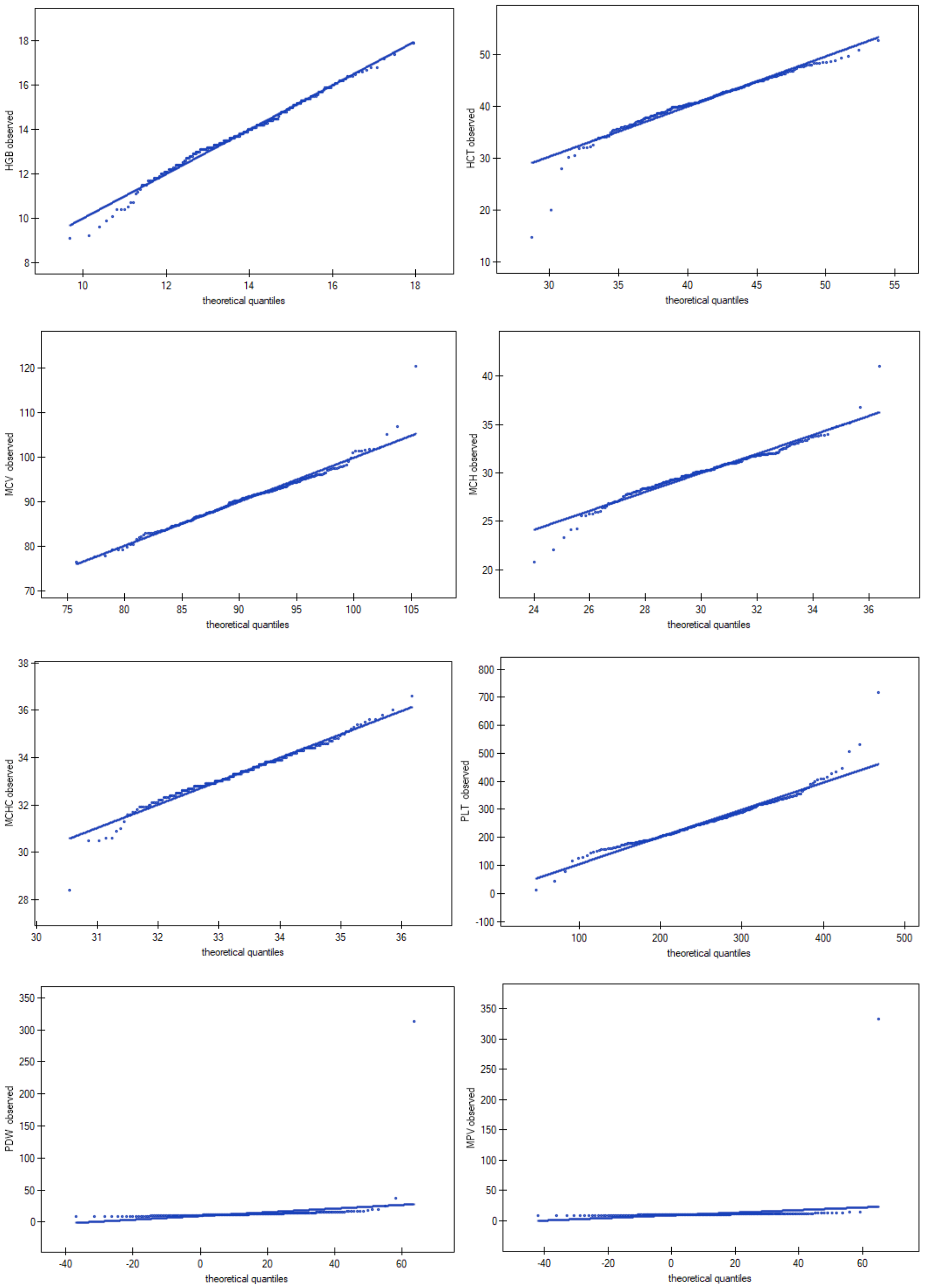

HGB observed
theoretical quantiles
HCT observed
theoretical quantiles
MCV observed
theoretical quantiles
MCH observed
theoretical quantiles
MCHC observed
theoretical quantiles
PLT observed
theoretical quantiles
PDW observed
theoretical quantiles
MPV observed
theoretical quantiles

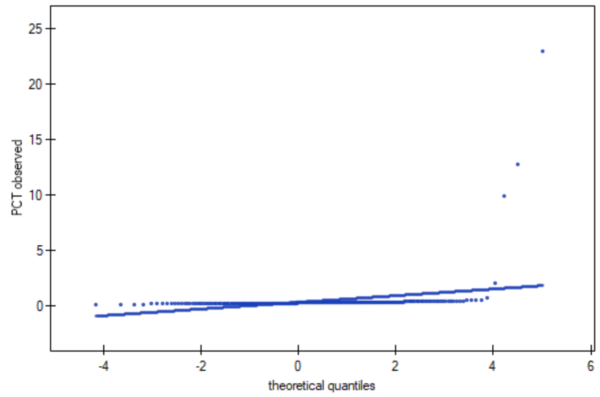
PCT observed
25
20
15
10
5
0
-4
-2
0
2
4
6
theoretical quantiles

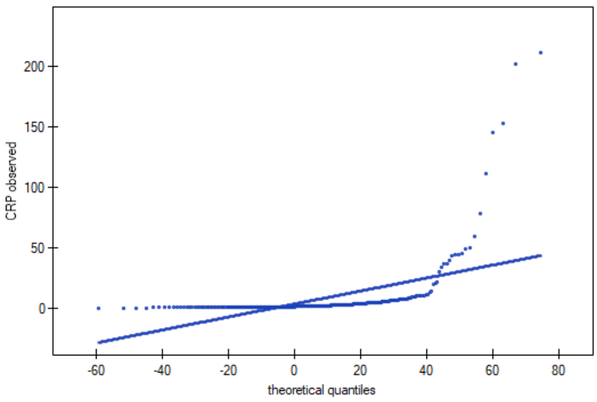
CRP observed
200
150
100
50
0
-60
-40
-20
0
20
40
60
80
theoretical quantiles

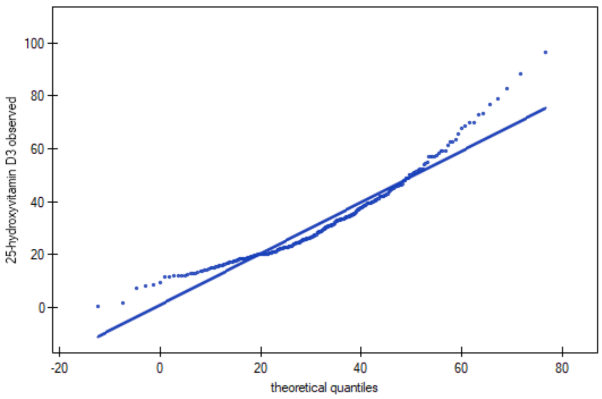
25-hydroxyvitamin D3 observed
100
80
60
40
20
0
-20
0
20
40
60
80
theoretical quantiles

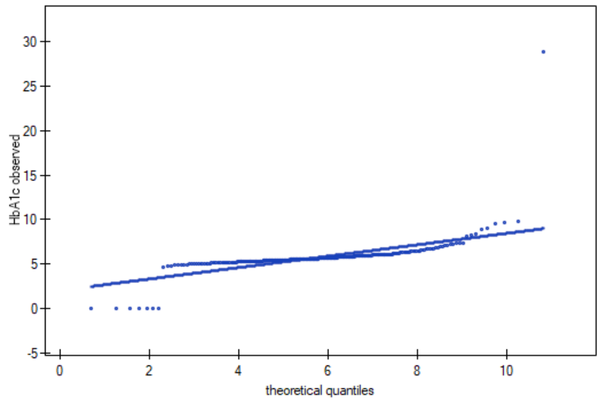
HbA1c observed
30
25
20
15
10
5
0
-5
0
2
4
6
8
10
theoretical quantiles

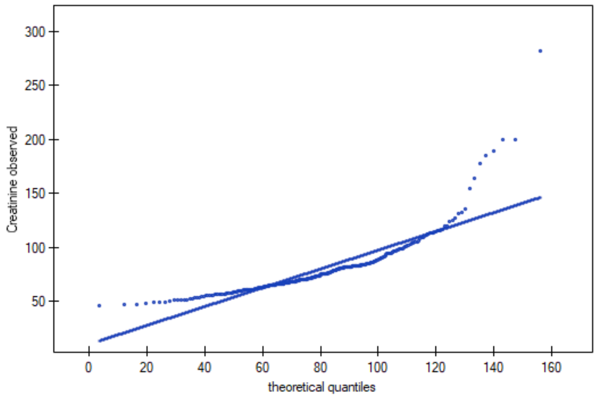
Creatinine observed
300
250
200
150
100
50
0
20
40
60
80
100
120
140
160
theoretical quantiles

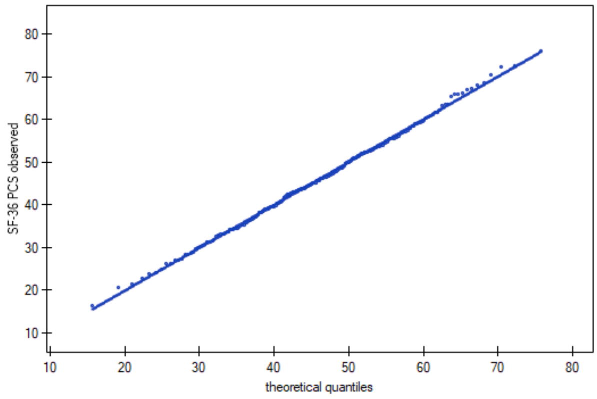
SF-36 PCS observed
80
70
60
50
40
30
20
10
10
20
30
40
50
60
70
80
theoretical quantiles

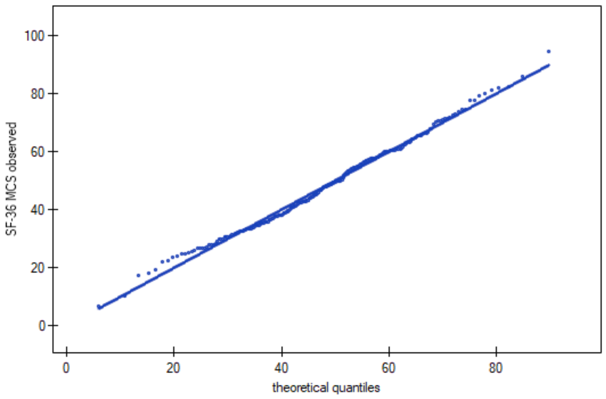
SF-36 MCS observed
100
80
60
40
20
0
0
20
40
60
80
theoretical quantiles

Figure S2. QQ-plots of variables after logarithmic transformation.

QQ-plots normally distributed variable, transformations by logarithm normalization: log Eosinophils (EOS, $\times 10^9$/L), log Basophils (BASO, $\times 10^9$/L), log Platelet distribution width (PDW, %), log Mean platelet volume (MPV, fL), log Plateletcrit (PCT, %), log C-reactive protein (CRP, mg/L), log 25-hydroxyvitamin D3 (ng/mL), log Glycated hemoglobin (HbA1c, %), log Creatinine (μmol/L)

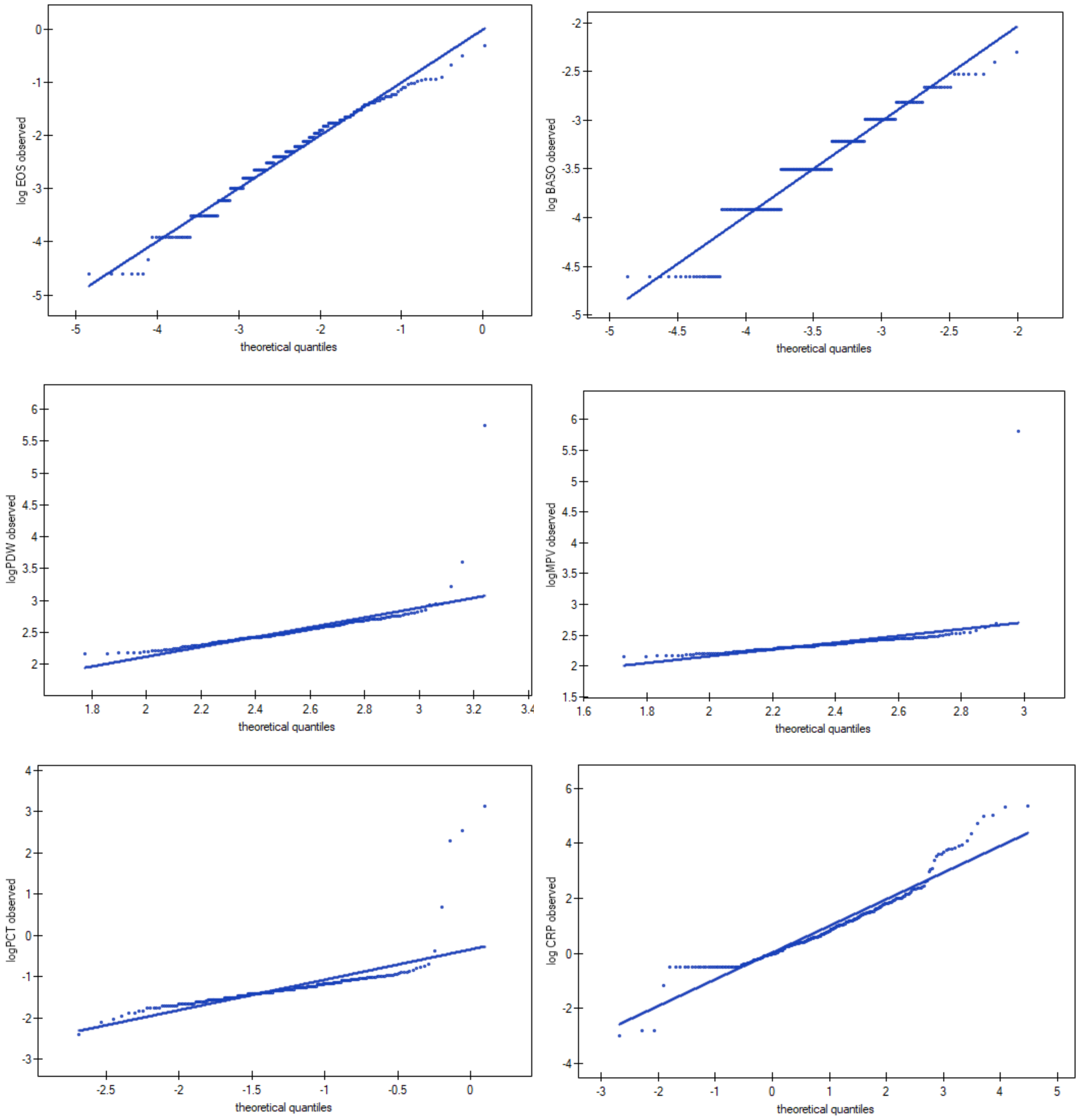

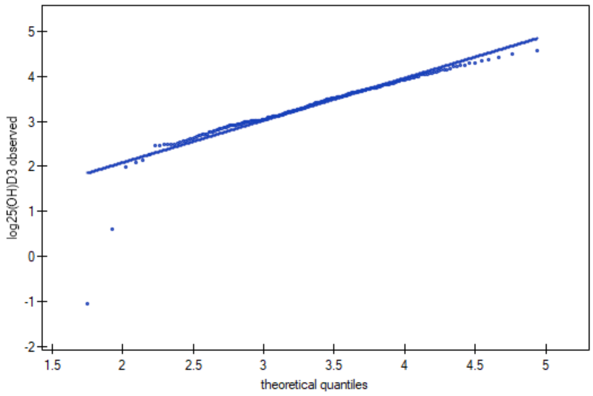
log25(OH)D3 observed
theoretical quantiles
1.5
2
2.5
3
3.5
4
4.5
5
-2
-1
0
1
2
3
4
5
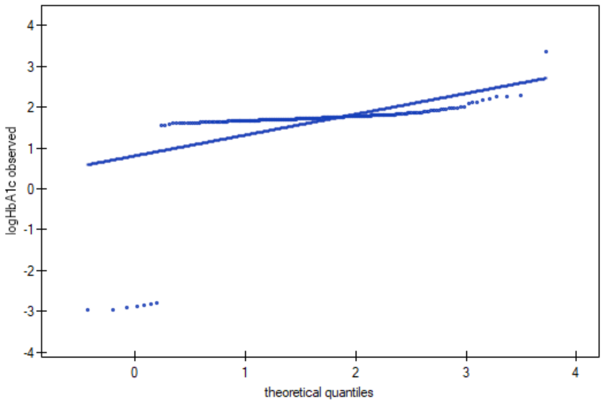
logHbA1c observed
theoretical quantiles
0
1
2
3
4
-4
-3
-2
-1
0
1
2
3
4
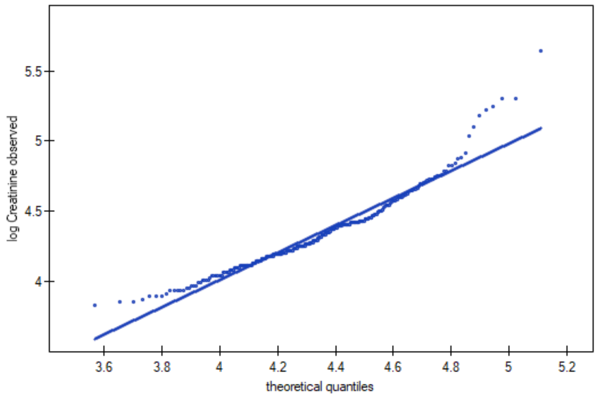
log Creatinine observed
theoretical quantiles
3.6
3.8
4
4.2
4.4
4.6
4.8
5
5.2
4
4.5
5
5.5
